# “Luke! Luke! Don’t! It’s a trap!”—spotlight on bias in animal experiments in nuclear oncology

**DOI:** 10.1007/s00259-020-04717-x

**Published:** 2020-02-10

**Authors:** Julie Nonnekens, Margret Schottelius

**Affiliations:** 1 Department of Molecular Genetics, Department of Radiology and Nuclear Medicine, Oncode Institute, Erasmus Medical Center Rotterdam, Rotterdam, Netherlands; 2 Translational Radiopharmaceutical Sciences, Department of Nuclear Medicine, Department of Oncology, Centre Hospitalier Universitaire Vaudois and University of Lausanne, Lausanne, Switzerland

Dear editor,

Animal experiments are vitally important for evaluation of newly developed nuclear tracers for imaging and therapy of cancers and cannot (yet) be adequately replaced. We have the ethical responsibility to keep the number of animals and level of discomfort to a minimum, but at the same time, we aim at generating preclinical data with maximum validity and statistical significance. The best way to balance these aspects is to design and perform these experiments *well*. However, a closer look at the current literature in the field of radiopharmaceutical development reveals a plethora of flaws and inconsistencies in the design and execution of preclinical animal experiments, challenging what we aim for most, i.e., validity of our data as an indispensable basis for clinical translation.

The validity of preclinical animal data is primarily defined by (A) the choice of an animal model with maximum achievable similarity in biology and symptoms with the human disease and (B) the choice of realistic, clinically translatable experimental conditions (e.g., dose regimens) [1]. Both aspects, in our opinion, are oftentimes not sufficiently well implemented, and we have written this letter to sharpen the awareness of the radiopharmaceutical research community for the existing bias in animal experiments and for the pitfalls leading to it. With due regard to our own areas of expertise, we explicitly limit the scope of our reflections to mouse models in nuclear oncology.

In this research area, it is particularly important to differentiate between the “natural” bias that is inherently connected with using mouse tumor models, and the bias that is introduced by choosing an inappropriate model for the evaluation of a novel radiopharmaceutical.

As to the first aspect, it is evident that a model, per definition, cannot be a perfect replication of the clinical condition [2]. We will always have to carefully consider the existing and undebatable (and sometimes unavoidable) differences between mice and men, with particular focus on the species dependence of (physiological) target expression as well as on a potential, sometimes very pronounced species selectivity of targeted tracers—a major source of bias in the interpretation of biodistribution data. Nonetheless, ongoing efforts are directed towards mimicking the development and biology of human cancers as closely as possible, and the researcher has the agony of choice between increasingly sophisticated syngeneic mouse tumor models, (human) cell-derived xenograft models and patient-derived xenograft (PDX) models. Each of them has their respective advantages and disadvantages with respect to their ability to reflect human disease as well as concerning their predictive value, ease of use, and cost-effectiveness [2]. These general issues invariably need to be taken into account and reviewed critically when selecting one or the other model for a given animal experiment in oncology.

However, preclinical radiopharmaceutical research is usually centered on *tracer* evaluation rather than *target* evaluation as in, e.g., classical oncological fundamental research studies trying to elucidate the role of a given molecular component in tumor biology or as a target for novel therapeutics. This entails a slightly different angle of view concerning the selection of an “appropriate” tumor model, and in this letter, we try to draw attention to some, in our opinion, crucial factors that challenge the validity of animal experiments in current nuclear oncology practice.

For example, it is widely established and generally accepted to use subcutaneous (human) xenograft models for tracer evaluation. In this context, validity of the resulting in vivo data is in large part determined by the target expression level on the cell line used for the induction of tumor growth. There is a vast spectrum of human or murine cancer cell lines available, which, being derived from actual tumors, by nature display realistic target expression levels and provide uptake data that allow a relatively accurate anticipation of tracer performance in patients, such as LNCaP prostate cancer cells for the evaluation for PSMA-targeted tracers. Nevertheless, there is a tendency towards employing transfected cell lines displaying 10- to 20-fold higher target expression (e.g., PC3 PIP in PSMA research). Of course, this generates eye-catchingly high absolute tumor uptake and striking tumor/non-tumor ratios, but generates data with no true predictive value for the human situation and leads to an overestimation of tracer performance [3, 4]. This issue has also been critically addressed in the context of the evaluation of novel ^18^F-labeled MCR1-targeted imaging agents [5] in a subcutaneous melanoma model with realistic MCR1 expression (human SK-MEL-1 cells with 1000 receptors/cell) compared with the historically used B16-F10 murine melanoma model (22.000 receptors/cell). Using two alternative ^18^F-labeled peptides with similar MCR1-affinity, but different molar activities (79 vs 193 MBq/nmol), the authors impressively demonstrate that *tracer optimization* is the key to a sensitive detection of low-level target expression. Since the sensitivity of target detection is a direct function of target expression, tracer affinity, and injected dose (molar activity), our efforts should be focused on optimizing tracers in these latter two respects. Furthermore, the use of “unrealistic” tumor models, unduly shifting the balance towards an apparent high sensitivity, should be abandoned for the sake of sincere tracer assessment.

Another aspect worth considering in this context is the currently established and accepted practice of investigating the influence of molar activity on tracer accumulation in target vs non-target tissues by injecting the tracer at a wide range of molar activities. When evaluating antibody-based tracers, this dose-finding process may be justified, although one might argue that the predictive value of these oftentimes extensive (> 40 mice) studies for clinical translation is highly limited, given the additional need for individual dose finding in clinical studies. Thus, for the sake of reducing animal numbers and based on the vast body of data already existing on radiolabeled antibodies, injecting 2–3 standard doses may also be sufficient for assessing the targeting performance of a given tracer.

Especially in the case of small-targeted agents such as PSMA ligands, however, PET dose-escalation studies are oftentimes performed solely on the grounds of generating optimal visual enhancement of tumor accumulation compared with PSMA-mediated kidney uptake—with the injected ligand doses mostly lying in a range with virtually no relation to clinically injectable doses [6]. For example, injection of 0.18 nmol of ligand (“intermediate molar activity”) in a 25-g mouse corresponds to 540 nmol (~ 550 μg, depending on the tracer used) in a 75 kg patient—a ligand dose beyond those used for PSMA-targeted radioligand therapy (~ 200 μg). At “low molar activities,” where tumor/kidney ratios finally reach values > 1, ligand amounts of 4.8–6 mg/patient are reached. This obvious lack of translatability of data and the inherent bias in these studies should, in our opinion, strongly encourage researchers in our field to critically reevaluate this current practice.

Another example, where metrics are in blatant disfavor of data validity, is the concept of radioligand therapy in mouse models. In therapy studies, the relevant parameter of observation is, by definition, the therapeutic efficiency of a given radiotracer. This, in turn, is determined by the dose deposited in the tumor—which, when considering the currently used protocols, is subject to substantial bias, just looking at simple mathematics: A tumor with 6 mm diameter (~ 1 g) represents a small tumor in a human and constitutes in average 1/75000 of the total body weight (TBW). In a mouse, the same tumor—and tumors frequently reach that size in therapy studies—corresponds to 1/25 of TBW. In humans, standard administered doses of, e.g., [^177^Lu]Lu-DOTA-TATE or [^177^Lu]Lu-PSMA-617 are in the range of 7.4 GBq/patient, corresponding to ~ 0.1 MBq/g body weight. Mice are injected with 30 MBq, leading to a more than tenfold higher ratio of activity/gram body weight, i.e., > 1 MBq/g. Given this substantial “overdosing” of mice compared with the human situation, it is not surprising that almost any radioligand therapy treatment has some effect on survival in mice, an outcome that is expected—and strongly biased. This, in our view, generally challenges the usefulness of radioligand therapy studies in mice, especially in the light of the excellent and constantly improving dosimetry extrapolations from mice to humans. Representative mouse biodistribution studies (applying realistic tracer doses) allow us to generate initial dosimetry data with good congruence with the human situation, and subsequent individual patient dosimetry, applying the therapeutic tracer at a low dose (< 185 MBq), provides much more valuable information on whole body dosimetry and the expected therapeutic efficiency of a given tracer than mouse therapy studies will ever do. We thus suggest to critically question our established workflows and to adjust them accordingly, driven as much by ethical considerations as by the desire to produce valid and valuable data.

In summary, we are well aware that all those involved in preclinical tracer development are doing their best for generating valid data with translational relevance. However, we do see room for improvement, not only on the experimental level but also on the level of peer review and Editorial Offices as well as Nuclear Medicine Societies. The community would certainly profit from a sharpened awareness concerning the “sense and nonsense” in our current practice of conducting animal experiments, including more critical review of obvious inconsistencies in the experimental setup and stricter quality control before publication. Moreover, a certain degree of harmonization concerning “approved” tumor models with realistic target expression or translatable tracer doses/molar activities, at least for one given molecular target, would greatly support a better comparability between data from different groups. And ultimately, it would allow us to reduce the number of bias “traps” on our way towards translating our developments into the clinic for the benefit of our patients.
